# The inherited methylome landscape is directly altered with paternal aging and associated with offspring neurodevelopmental disorders

**DOI:** 10.1111/acel.13178

**Published:** 2020-07-01

**Authors:** Michelle M. Denomme, Mary E. Haywood, Jason C. Parks, William B. Schoolcraft, Mandy G. Katz‐Jaffe

**Affiliations:** ^1^ Fertility Labs of Colorado Lone Tree CO USA; ^2^ Fertility Genetics Lone Tree CO USA; ^3^ Colorado Center for Reproductive Medicine Lone Tree CO USA

**Keywords:** advanced paternal age, blastocyst, DNA methylation, epigenetics, offspring neurodevelopmental disorders, sperm

## Abstract

Paternal aging and the prevalence of neurodevelopmental disorders in offspring are well documented. Yet, the underlying mechanism and the mode of inheritance have not been conclusively established. Advancing paternal age is a subtle and varying phenotype. As such, it is likely that a threshold for cumulative risk may exist that, if surpassed, culminates in a predisposition to disease and ultimately an observed phenotype in offspring. Epigenetic regulation provides a plausible explanation for the nongenetic paternal transmission of disease susceptibility. With the use of whole‐genome methylation sequencing, the data described herein substantiate an increasingly compromised DNA methylation profile as sperm ages and, for the first time, also demonstrate a generational correlation in sperm and blastocyst of an altered methylome associated with advanced paternal age. Methylation alterations are not randomly distributed across the genome, but appear clustered at certain chromosomal locations, and significantly colocalize with regions of nucleosome retention. Genes associated with autism spectrum disorder, schizophrenia, and bipolar disorder are significantly enriched with causative methylation aberrations in both sperm and embryos from aged fathers. The long‐term health burden and societal economic impact of these conditions are substantial and will continue with increasingly prevalent diagnosis. This work provides a mechanistic link between the paternal age effect and offspring neurodevelopmental disorders leading to a better understanding of causation and investigation into potential future therapy.

## Graphical abstract

A generational epigenetic correlation was observed for human sperm and blastocyst methylomes in association with advanced paternal age at genes implicated in offspring neurodevelopmental disorders, including autism spectrum disorder, schizophrenia, and bipolar disorder. This work provides a mechanistic link between the paternal age effect and offspring neurodevelopmental disorders leading to a better understanding of causation and clinical management.

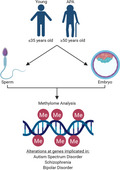

## 1 INTRODUCTION

The influence of advanced paternal age (APA) on adverse offspring health has been well documented, including increased disease risk of neurodevelopmental disorders (Frans et al., 2008; Hare & Moran, 1979; Miller et al., 2011). A recent meta‐analysis of paternal age and psychiatric disorders indicated significantly elevated risk estimates for children conceived by fathers over 50 years, with adjusted odds ratio (OR) for developing autism spectrum disorder ranging from 2.26 to 3.37, schizophrenia OR 1.59–3.80, and bipolar disorder OR 1.27–2.84, compared with children conceived by young fathers (de Kluiver, Buizer‐Voskamp, Dolan, & Boomsma, 2017). The long‐term health burden of these conditions for the affected children, as well as the economic impact on their families and to society, demands a better understanding of the causative link between paternal age and offspring neurodevelopmental disorders.

The causal mechanism underlying this paternal age effect has not been elucidated, nor has the mode of inheritance yet to be conclusively established. Observed phenotypes derived by mammalian non‐Mendelian genetics suggest that factors independent of DNA sequence play a significant role (Bassett, Chow, O'Neill, & Brzustowicz, 2001). Rather, modifiable and inheritable epigenetic information, such as DNA methylation, has this potential to be generationally transmitted. A series of epigenetic reprogramming events occur during gametogenesis and immediately after fertilization (Reik, Dean, & Walter, 2001). Erasure and gamete‐specific establishment of methylation marks occur during primordial germ cell development. Directly after fertilization, the male pronucleus undergoes a rapid, active demethylation process, while the female pronucleus undergoes passive replication‐dependent demethylation, prior to re‐establishment in the developing embryo. Imprinted genes, and perhaps other developmentally critical regions, elude the embryonic portion of epigenetic reprogramming, enabling epigenetic generational inheritance (Kobayashi et al., 2012). A longitudinal paired study of sperm collected one‐to‐two decades apart demonstrated age‐associated DNA methylation alterations in the paternal germline, with a portion of these changes located at genes associated with schizophrenia and bipolar disorder (Jenkins, Aston, Pflueger, Cairns, & Carrell, 2014). The impact of aging on the sperm methylome was also confirmed in a mouse model, with subsequent age‐associated methylation alterations observed in murine offspring, influencing behavioral phenotypes (Milekic et al., 2015).

Unlike genetic errors, epigenetic information can be disrupted by various external environmental and endogenous factors during reprogramming events, which could be inherited and lead to long‐term adverse consequences affecting offspring health (Kimura, Yoshizaki, & Osumi, 2018). Exposure to famine in utero, like the Dutch Hunger Winter, resulted in epigenetic silencing of certain genes in offspring, causing higher rates of obesity, diabetes, schizophrenia, and mortality (Lumey, Stein, & Susser, 2011; Lumey et al., 2012; Tobi et al., 2018). Epigenetic dysregulation is also prevalent in cancer development (Franco, Schoneveld, Georgakilas, & Panayiotidis, 2008). Oncogenesis is commonly associated with global hypomethylation, inducing genomic instability and activation of oncogenes, as well as promoter hypermethylation leading to inactivation of tumor suppressor genes (Kulis & Esteller, 2010). Notably, an increased incidence of pediatric cancers has been implicated in offspring of older fathers (Hemminki, Kyyronen, & Vaittinen, 1999; Yip, Pawitan, & Czene, 2006). The process of aging can modulate epigenetic regulation, in both somatic cells and the germline. Previously published work has confirmed these effects on the sperm methylome over time (Jenkins et al., 2014). Inherited epigenetic alterations in older males may be the mechanism leading to the increased disease susceptibility in their offspring.

Our data substantiate a compromised DNA methylation profile in aged sperm, supporting the hypothesis of incomplete reprogramming during spermatogenesis. For the first time, we also demonstrate a generational correlation in sperm and embryo of an altered human methylation landscape associated with advanced paternal age, contributing to nonequivalent efficiency of methylation re‐establishment throughout the human blastocyst genome. Aberrant epigenetic reprogramming is significantly enriched at genes essential for neurological development in both APA sperm and blastocysts, and provides a mechanistic link between the paternal age effect and offspring neurodevelopmental disorders.

## 2 RESULTS

### 2.1 Patient demographics

Advanced paternal age was defined as ≥ 50 years, while young men ≤ 35 years were selected as controls. Demographic data for all male participants are presented in Table S1. Only normozoospermic samples were utilized based on WHO criteria (Cooper et al., 2010) and/or internal clinical standards. In addition, only blastocysts donated from young fertile donor oocyte in vitro fertilization (IVF) cycles were utilized in order to effectively eliminate known female and male factor infertility.

### 2.2 Sperm and blastocyst methylomes

We used a modified reduced representation bisulfite sequencing (RRBS) method for global methylome analysis on six young (average age: 28.5 ± 2.1 years) and six APA (average age: 54.5 ± 3.9 years) normozoospermic sperm samples, achieving an average of 32 million read pairs and 46% mapping efficiency. In total, we captured approximately 10 million unique CpG sites at 8X coverage, consistent among genomic features: gene body (85%), promoter (73%‐76%), and CpG islands (77%‐81%) (Table S2). An increase in global methylation was observed in the APA sperm epigenome and confirmed by *LINE1* bisulfite pyrosequencing (Figure S1).

Due to the extremely limited starting material, a whole‐genome bisulfite sequencing (WGBS) approach was required for global methylome analysis of six young (average paternal age: 32.3 ± 1.5 years) and six APA (average paternal age: 53.5 ± 3.4 years) blastocyst samples. An average of 480 million read pairs and 62% mapping efficiency was achieved, capturing 34 million unique CpGs at 25X coverage. With limited starting input, genomic coverage was moderately adversely affected; gene body coverage was high and consistent (85%‐89%), followed by promoters (69%‐84%) and CpG islands (56%‐78%) with variable coverage across samples (Table S2). At 5X coverage, 654,665 CpGs were common to both sperm and blastocysts.

**Figure 1:**
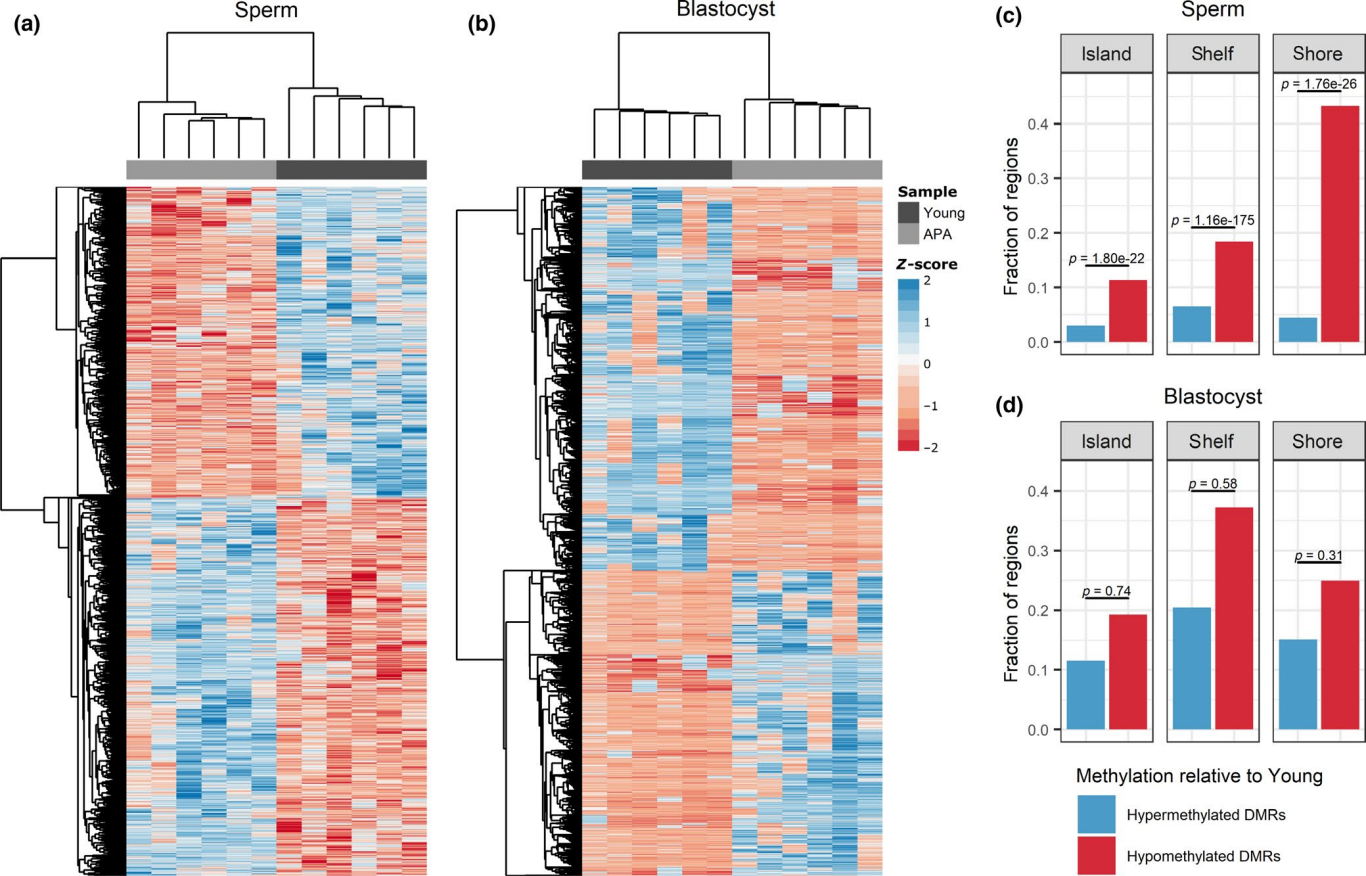
Significant DMR‐associated CpG sites and the fraction of DMRs localized to CpG islands, shelves, and shores. (a–b) Heat map representation of the hierarchical clustering of significant (*p* ≤ .05) DMR‐associated CpG sites in a) sperm (*n* = 15,991 CpGs) and b) blastocyst (*n* = 14,461 CpGs), where a positive z‐score corresponds to hypermethylation and a negative z‐score corresponds to hypomethylation relative to the mean. Samples in both sperm and blastocyst cluster into two distinct groups by young and APA samples. (c–d) The proportion of DMRs associated with CpG islands, shelves, and shores in c) sperm and d) blastocyst was tallied for hypermethylated and hypomethylated DMRs in APA relative to young. Hypomethylated CpG regions were significantly enriched in sperm but not blastocyst

We identified 49,722 and 106,995 CpGs as statistically significant between the young and APA groups for sperm and blastocysts, respectively (*p* ≤ .05). The use of a sliding window analysis reduced the risk of false positives by calling differentially methylated regions (DMRs) with ≥ 3 significantly altered CpGs in the same direction within a 1KB region. This analysis resulted in 3,405 sperm DMRs and 3,997 blastocyst DMRs (Table 1). Unsupervised hierarchical clustering analysis of significant DMR‐associated CpGs differentiated between the young and APA groups in both sperm and blastocysts, demonstrating distinct sample branches and unique DMR methylation patterns (Figure 1a‐b). Hypomethylated DMRs in APA sperm were significantly enriched at CpG islands (*p* = 1.80 × 10^‐22^), shelves (*p* = 1.16 × 10^‐175^), and shores (*p* = 1.76 × 10^‐26^), with a similar trend observed preferentially for hypomethylated DMRs in APA blastocysts, although not to significance (Figure 1c‐d).

**Table 1:** APA sperm and blastocyst DMRs

	APA sperm (*n* = 3,405 DMRs) Paternal age: Young (28.5 ± 2.1 years) APA (54.5 ± 3.9 years)		APA blastocyst (*n* = 3,997 DMRs) Paternal age: Young (32.7 ± 1.4 years) APA (52.7 ± 3.0 years)	
	Hypermethylated	Hypomethylated	Hypermethylated	Hypomethylated
Total DMRs	1929	1,476	1729	2,268
Significant CpGs in DMRs	8,729	7,262	6,217	8,244
Average CpGs per DMR	4.5 (range 3–35)	4.9 (range 3–40)	3.6 (range 3–25)	3.6 (range 3–14)
Average DMR window width	211 bp (range 3–4069)	370 bp (range 3–3792)	550 bp (range 3–5432)	544 bp (range 4–4033)
Total associated genes	612	810	1,087	1,457
Average genes per DMR	0.32	0.55	0.63	0.64
Total unique associated genes	568	766	803	1,173
DMRs not associated with a gene	1,291 (67%)	641 (43%)	428 (25%)	497 (22%)

A statistical comparison of the sperm and blastocyst DMR‐associated genes demonstrated a highly significant enrichment of genes between the two methylomes upon paternal aging (*p* = 3.46 × 10^‐55^; odds ratio [OR] 3.19). We identified 218 genes with significant and directional DMRs, 167 genes were hypomethylated, and 61 genes were hypermethylated in both sources, with 10 genes exhibiting both hypo‐ and hypermethylated DMRs (Table 2).

**Table 2:** Overlapping DMRs

	APA sperm‐blastocyst overlapping DMRs		
	Hypermethylated	Hypomethylated	Any
Overlapping DMR‐associated genes	61	167	323
Fold enrichment (odds ratio)	2.36	4.31	3.19
OR 95% confidence interval	1.77–3.10	3.58–5.17	2.79–3.65
*p*‐value	2.25E−08	6.14E−45	3.46E−55

**Figure 2:**
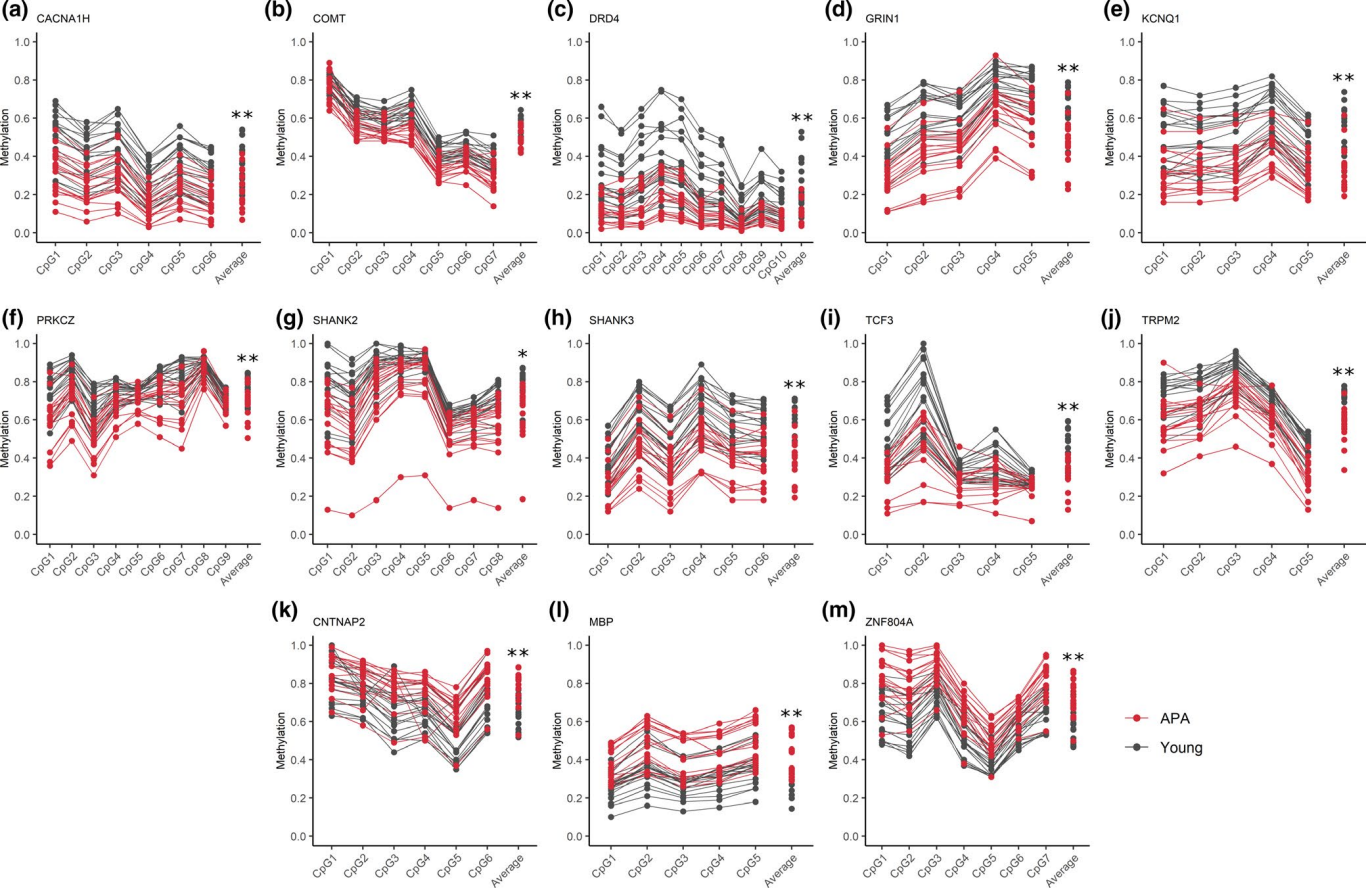
Sperm DNA methylation validation results. Each plot represents pyrosequencing results for selected validation genes at distinct CpG sites within a significant DMR. CpG sites on the x‐axis are followed by the average percent methylation across all CpGs for young (gray) and APA (red) individuals. Each line represents results for individual sperm samples (*n* = 18 young and *n* = 18 APA sperm samples). Significant hypomethylation in APA relative to young is demonstrated in plots (A‐J), and significant hypermethylation in APA relative to young is demonstrated in plots (K‐M); *p ≤ .01, **p ≤ .001

**Figure 3:**
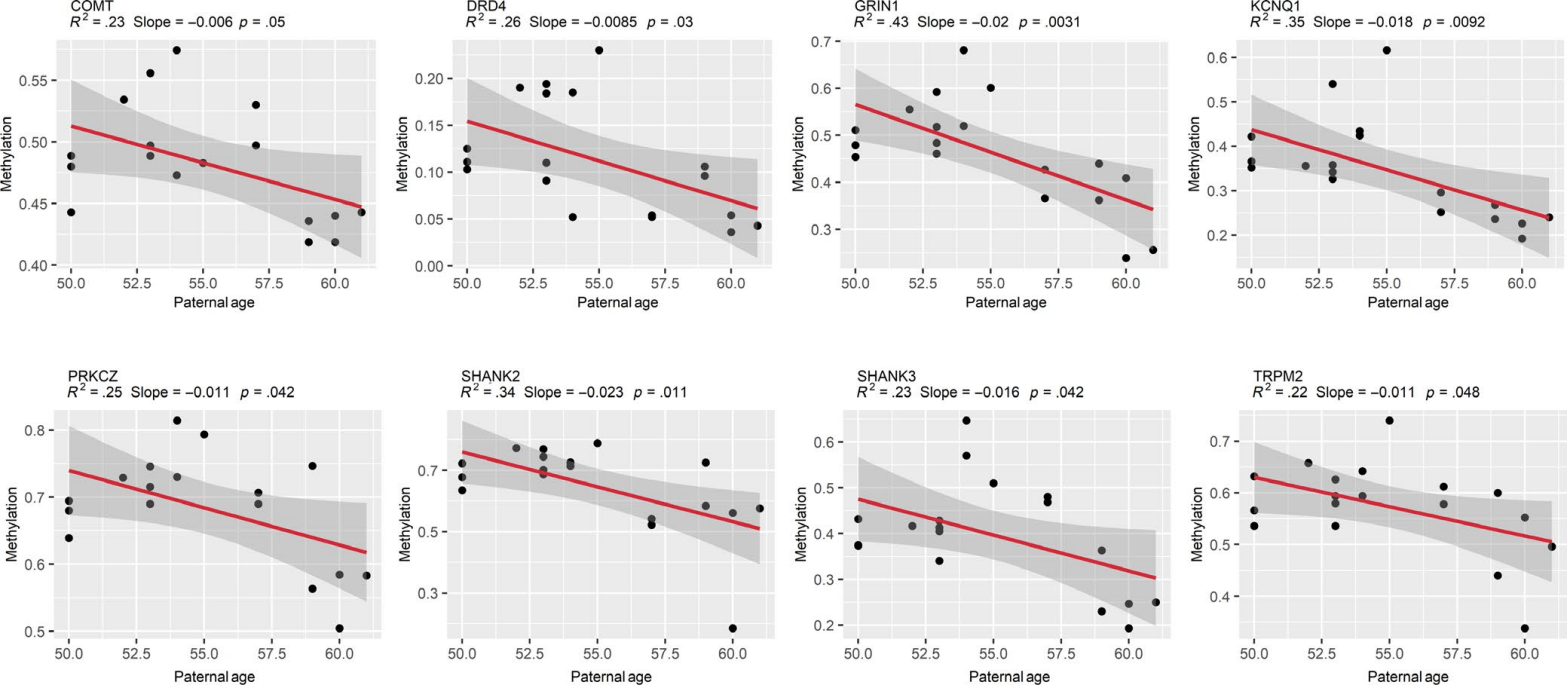
Sperm DNA methylation change by paternal age. Linear regression models demonstrate a significant negative association between sperm DNA methylation and paternal age in APA fathers (≥50 years; *n* = 18 APA sperm samples). Models were fitted using the lm() function in R with default arguments, and those with *p* ≤ .05 were considered significant. R‐squared, slope, and p‐values are displayed above each plot, and shaded gray areas around each red regression line represent a 95% confidence interval

Methylation validation was achieved in an independent cohort of twenty‐four sperm samples from additional young (*n* = 12; average age: 30.2 ± 1.5 years) and APA (*n* = 12; average age: 55.3 ± 3.7 years) normozoospermic men (*p* < .05; Figure 2). Genes were selected for methylation validation for various reasons, including altered DMRs from both sperm and blastocyst methylomes, genes identified in significant pathways, genes implicated in neurodevelopmental disorders, genes localized to significant cytobands, imprinted genes, or genes that correspond with published literature on aging sperm (Jenkins et al., 2014) (Table S3). We generated linear regression models using the APA sperm results to further characterize methylation changes with respect to aging. As paternal age increased from 50 years to > 60 years, hypomethylation was observed to be significantly exacerbated (Figure 3). DMRs from two genes significantly associated with neurodevelopmental disorders were selected for blastocyst methylome validation. For this, we used a cohort of twelve blastocysts derived from young paternal age fathers (average paternal age: 31.1 ± 2.2 years) and twelve APA blastocysts (average paternal age: 55.3 ± 3.1 years). Pyrosequencing data confirmed significant hypomethylation along the amplified *CACNA1H* DMR region, with 40% average methylation for the young paternal age blastocyst group significantly decreased to 25% in the APA blastocyst group (*p* < .05), while validation of the *SHANK2* blastocyst DMR showed a trend toward hypomethylation (young: 75%, APA: 56%; *p* < .1) (Figure S2).

### 2.3 Localization of DMRs

**Figure 4:**
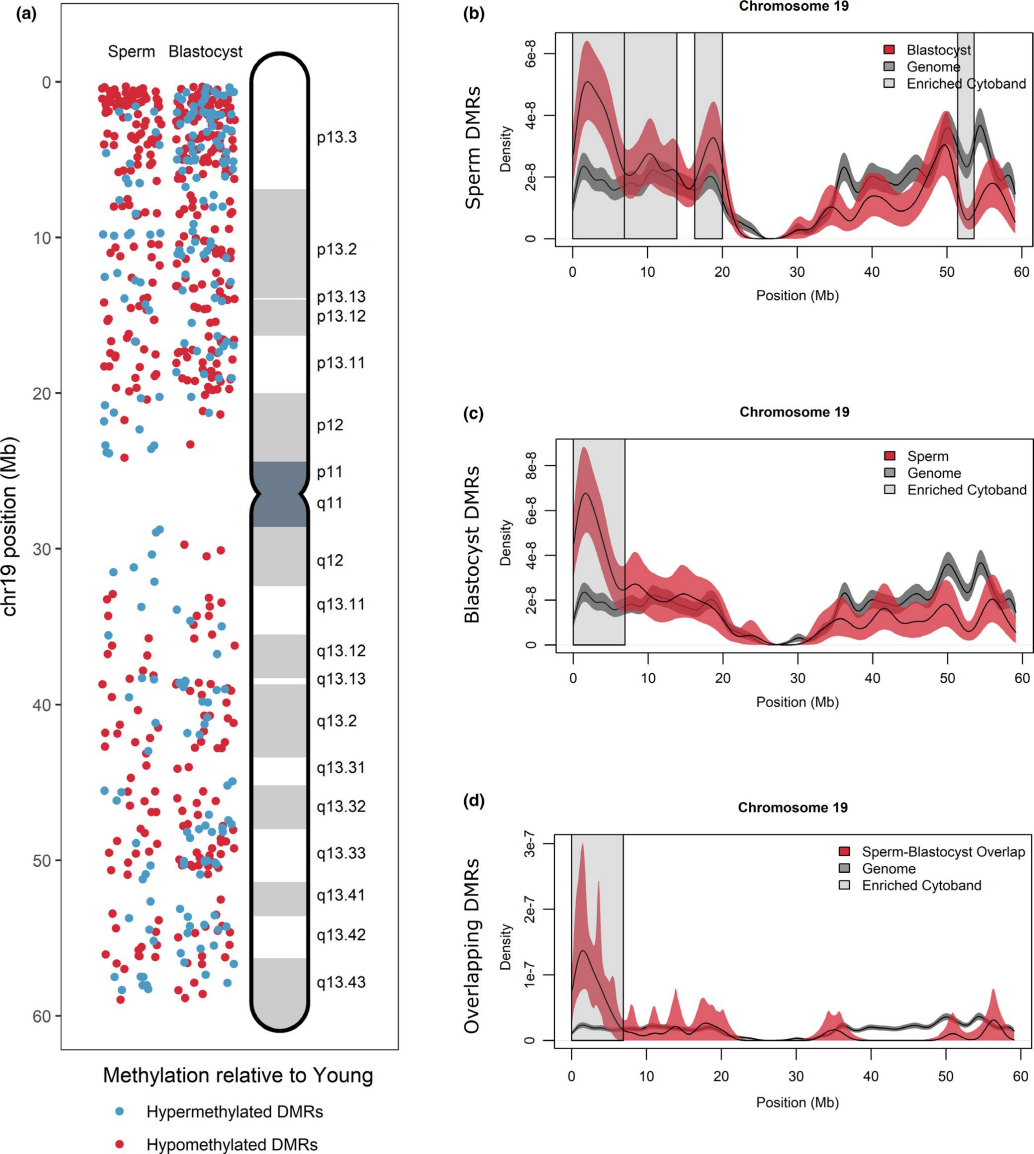
DMR localization on Chromosome 19. (a) DMRs on chromosome 19 were localized for sperm (left panel) and blastocyst (right panel) according to hypermethylated DMRs (blue) and hypomethylated DMRs (red) in APA relative to young. Increased DMR clustering is observed for cytoband 19p13.3. (b–d) All genes located on chromosome 19 (gray lines) were compared with DMR‐associated genes (red lines) in (b) sperm, (c) blastocyst, and (d) overlapping sperm blastocyst using kernel density estimates. Shaded areas around the lines signify the 95% confidence interval. Cytobands where the density of genes was significantly different between DMR‐associated genes and the genome are highlighted with gray boxes and include cytoband 19p13.3 for all comparisons

To determine whether specific chromosomal regions were more susceptible to age‐related methylation alterations, we analyzed chromosomal enrichment for DMR‐associated gene density at individual cytobands (*p* < .05, *q* < 0.05). We found that methylation alterations were not randomly distributed across the genome, but appear clustered at certain chromosomal locations, with chromosome 19 having the greatest significance between young and APA samples (sperm: *p* = 5.51 × 10^‐7^, *q* = 6.62 × 10^‐6^, blastocyst: *p* = 9.01 × 10^‐13^, *q* = 1.08 × 10^‐11^, and overlapping: *p* = 7.28 × 10^‐6^, *q* = 1.75 × 10^‐4^, OR: 2.21) (Figure 4a). A significant enrichment was identified at 5 cytobands in APA sperm. Four of the five cytobands were independently enriched in the APA blastocyst methylome, and three cytobands were significant in the overlapping APA sperm and blastocyst gene list (Table S4). The greatest enrichment for all three datasets was chr19p13.3 (sperm: *p* = 4.32 × 10^‐19^, blastocyst: *p* = 3.76 × 10^‐22^, and overlapping: *p* = 5.62 × 10^‐16^, OR: 9.89) (Figure 4b‐d).

We subsequently analyzed the colocalization of DMR‐associated genes with known regions of nucleosome retention in sperm, using previously published ChIP data by Hammoud and colleagues (Hammoud et al., 2009). For APA sperm DMRs, a statistically significant enrichment was identified with mononucleosomes (*p* = 9.36 × 10^‐29^; OR: 1.89) and with the presence of the repressive histone mark H3K27me3 (*p* = 7.21 × 10^‐6^; OR: 1.38). The colocalization with mononucleosomes was enhanced for the directionally overlapping DMRs identified in both sperm and blastocyst datasets (*p* = 2.52 × 10^‐18^; OR: 3.32) (Table S5).

### 2.4 Pathway analysis and disease associations

Several neurological signaling pathways were highly represented among regions of the genome that were differentially methylated in our APA samples. In particular, the opioid signaling pathway was the top canonical pathway for the directionally overlapping genes found in both APA sperm and blastocyst datasets (*p* = 2.14 × 10^‐3^). This pathway was also the most significant pathway identified independently for the APA blastocyst methylome (*p* = 2.48 × 10^‐7^), and the fourth pathway identified for the APA sperm methylome (*p* = 1.24 × 10^‐3^). Multiple genes from the opioid signaling pathway were successfully validated in APA sperm (*CACNA1H*, *GRIN1*, and *PRKCZ*) and APA blastocysts (*CACNA1H*). The pathway involving nNOS signaling in neurons was highlighted as the top canonical pathway impacted in the APA sperm methylome (*p* = 2.08 × 10^‐4^), which also comprises similar successfully validated genes (*GRIN1* and *PRKCZ*). Additional pathways impacted by advanced paternal age can be found in Table 3.

**Table 3:** Top canonical pathways

Rank	Pathway	*p*‐value
APA sperm DMRs		
1	nNOS signaling in neurons	2.08E−04
2	Sumoylation pathway	2.50E−04
3	Glutamate receptor signaling	2.54E−04
4	Opioid signaling pathway	1.24E−03
5	Dopamine‐DARPP32 feedback in cAMP signaling	4.17E−03
APA blastocyst DMRs		
1	Opioid signaling pathway	2.45E−07
2	Netrin signaling	3.58E−07
3	GPCR‐mediated nutrient sensing in enteroendocrine cells	1.69E−06
4	CREB signaling in neurons	4.28E−06
5	PPARα/RXRα Activation	5.77E−06
APA sperm‐blastocyst directional overlapping DMRs		
1	Opioid signaling pathway	2.14E−03
2	Tight junction signaling	4.84E−03
3	Wnt/β‐catenin signaling	5.42E−03
4	Role of Wnt/GSK−3β signaling in the pathogenesis of influenza	5.71E−03
5	Phosphatidylethanolamine biosynthesis III	9.26E−03

To determine whether paternal aging alters methylation at genes linked to neurodevelopmental disorders, we compared publicly available gene lists to our DMRs. We identified a highly significant enrichment for genes implicated in three neurodevelopmental disorders for the directionally overlapping DMRs, specifically for autism spectrum disorder (*p* = 1.94 × 10^‐4^; OR: 4.34), schizophrenia (*p* = 5.55 × 10^‐4^; OR: 5.30), and bipolar disorder (*p* = 4.73 × 10^‐5^; OR: 3.30). This enrichment was also identified independently in the APA sperm DMRs and APA blastocyst DMRs (Table 4). Several genes were validated in APA sperm that were associated with autism spectrum disorder (*CACNA1H*, *CNTNAP2*, *GRIN1*, *SHANK2*, *SHANK3*, *ZNF804A*), schizophrenia (*TCF3*, *ZNF804A*), and bipolar disorder (*COMT*, *DRD4*, *GRIN1*, *MBP*, *PRKCZ*, *SHANK2*, *TRPM2*, *ZNF804A*), and in APA blastocysts (*CACNA1H*). Notably, the opioid signaling pathway is shared by all three candidate neurodevelopmental disease gene lists (Table S6).

**Table 4:** Neurodevelopmental disorder associations

Disease	Overlapping genes	Fold enrichment (odds ratio)	OR 95% confidence interval	*p*‐value
APA sperm DMRs				
Autism spectrum disorder	35	2.48	1.68–3.57	7.76E−06
Schizophrenia	19	2.31	1.35–3.77	1.57E−03
Bipolar disorder	63	1.93	1.45–2.53	8.24E−06
APA blastocyst DMRs				
Autism spectrum disorder	60	2.80	2.06–3.76	2.88E−10
Schizophrenia	40	3.40	2.30–4.94	2.42E−09
Bipolar disorder	91	1.71	1.35–2.16	1.17E−05
APA sperm‐blastocyst directional overlapping DMRs				
Autism spectrum disorder	10	4.34	2.03–8.28	1.94E−04
Schizophrenia	7	5.30	2.07–11.38	5.55E−04
Bipolar disorder	17	3.30	1.87–5.47	4.73E−05

To further characterize our findings with respect to neurodevelopmental disorders, we utilized the publicly available methylation profiling array described by Nardone and colleagues (Nardone et al., 2014). We compared two brain tissues between autistic and nonautistic samples and identified 141 and 62 DMRs, respectively (Table 5). We found that the genes within these DMRs significantly overlapped our APA sperm (frontal cortex *p* = 5.78x10^‐9^, cingulate cortex *p* = 1.62x10^‐4^) and APA blastocyst (frontal cortex *p* = 1.94 × 10^‐16^, cingulate cortex *p* = 3.24 × 10^‐5^) DMR‐associated gene lists. Using the genes associated with these DMRs, we performed pathway analysis and found that the frontal cortex was also significantly enriched for opioid signaling (*p* = 2.14 × 10^‐3^; Table 6).

**Table 5:** DMR‐associated genes in autistic brains compared with APA sperm and APA blastocyst

Nardone et al., 2014	DMRs	Genes in DMRs	APA sperm gene overlap	*p*‐value	APA blastocyst gene overlap	*p*‐value
Autistic frontal cortex	141	86	22	5.78E−09	38	1.94E−16
Autistic cingulate cortex	62	42	10	1.62E−04	14	3.24E−05

**Table 6:** Pathway analysis for DMR‐associated genes in autistic brains compared with APA sperm and APA blastocyst

Canonical pathway (*p* ≤ .01)	Autistic frontal cortex (Nardone et al., 2014)^23^	APA sperm	APA blastocyst
Opioid signaling Pathway	2.14E−03	1.23E−03	2.45E−07
Melatonin signaling	2.24E−03	6.17E−03	3.72E−04
Dopamine‐DARPP32 feedback in cAMP signaling	2.88E−03	4.17E−03	1.10E−05
Neuregulin signaling	5.13E−03	5.25E−03	*N*.S.

Given that genomic imprints are established in the germline and can persist through fertilization, along with their critical functions in the developing brain and various overlapping characteristics with neurodevelopmental disorders, it is reasonable to consider their involvement in mediating APA effects. The APA datasets were therefore compared to the current list of known and putative human‐imprinted genes. We found that APA sperm had 19 hypomethylated and 7 hypermethylated significant DMRs that were associated with imprinted genes. Blastocysts derived from APA fathers revealed 22 hypomethylated and 10 hypermethylated significant DMRs associated with imprinted genes, and 6 imprinted genes overlapped in both datasets (*p* ≤ .05; Table S7).

### 2.5 Sperm miRNA profiles

The same six young (average age: 28.5 ± 2.1 years) and six APA (average age: 54.5 ± 3.9 years) normozoospermic sperm samples used for global methylome analysis were also used for small RNA sequencing, identifying 278 expressed miRNAs with > 5 counts per million. However, the expression was consistent between the two groups with no differentially expressed miRNAs following FDR adjustment (*p* ≤ .05, q ≤ 0.05; Figure S3). Sixteen DMRs were situated near genes that encode for miRNAs, with no significant impact to miRNA expression (Table S8).

## 3 DISCUSSION

Risks of offspring neurodevelopmental disorders are increased with advancing paternal age (Croen, Najjar, Fireman, & Grether, 2007; Frans et al., 2008; Malaspina et al., 2002). One potential mechanism involves epimutations arising over time in the sperm, resulting in a phenotype that then manifests later in offspring development (Milekic et al., 2015). We are the first to report an observed generational inheritance of epigenetic dysregulation from human sperm to the preimplantation embryo. Our results demonstrate a mechanism for the paternal age effect from sperm to offspring, with confirmed significant susceptibility at neurodevelopmental genes associated with autism spectrum disorder, schizophrenia, and bipolar disorder. Furthermore, the genes identified in our study significantly overlapped those that were also differentially methylated in the frontal cortex and cingulate cortex of autistic brains (Nardone et al., 2014).

In accordance with the literature on aging sperm methylation (Jenkins, Aston, Cairns, & Carrell, 2013; Jenkins et al., 2014), an increase in global methylation was observed in the APA sperm epigenome; however, our DMR analysis identified a comparable number of hypermethylated and hypomethylated regions. It was striking that, among individuals, these methylation changes occurred with tight consistency at each CpG site of validated genes, seen in both the original and independent sperm cohorts. Additionally, linear regression analysis clearly illustrated significant progressive methylation alterations as paternal age increased. Interestingly, while considerable DNA methylation changes were identified between the young and APA sperm samples, differential expression of small RNAs was not observed, suggesting that miRNA regulation is not an epigenetic mechanism affected by advancing paternal age.

There was a highly significant overlap of DMR‐associated genes between the sperm and blastocyst methylomes upon paternal aging, suggesting that the same genomic regions are affected by methylation dysregulation. Methylation alterations in our datasets were not randomly distributed across the genome, but appear clustered at certain chromosomal locations. Subtelomeric regions were highly enriched for methylation alterations, particularly cytoband 19p13.3, for both sperm and blastocyst. This cytoband harbors a large number of genes (second only to cytoband 16p13.3, also significant in all three groups). Access to these genes may require a looser chromatin conformation, generating vulnerability to epigenetic alterations. It has also been proposed that methylation in subtelomeric regions like these may escape the large‐scale epigenetic reprogramming events and therefore have potential susceptibility to disruption and transmission to offspring (Guibert, Forne, & Weber, 2012; Hajkova et al., 2002; Popp et al., 2010), and age‐associated effects have previously been localized to these regions in sperm (Jenkins et al., 2014).

This observation that methylation alterations were not randomly distributed leads to the hypothesis that particular features of chromatin packaging in APA sperm are vulnerable to epigenetic errors, giving rise to aberrant reprogramming within the same regions in blastocysts. As such, we postulated that the DMRs identified in APA sperm colocalized with regions of retained histones. During spermiogenesis, the majority of histones are exchanged for protamines, but as many as 15% of nucleosomes are retained in regions of high CpG density and enriched at loci of developmental importance, including developmental gene promoters, imprinted loci, and genes encoding miRNAs (de Vries et al., 2013; Hammoud et al., 2009). By comparing our findings to those putatively bound to retained histones, previously identified by ChIP data from Hammoud et al., 2009, a statistically significant enrichment of APA sperm DMR‐associated genes correlated with nucleosome retention. A comparable enrichment was observed for the directional overlapping DMRs identified in both sperm and blastocyst datasets. This suggests that paternal germline methylation alterations induced by advancing age is transmitted in a chromatin context. In particular, hypomethylated DMRs were more likely to be associated with a gene, to be associated with CpG islands and flanking regions, and more likely to colocalize with mononucleosomes. Interestingly, while our DMR‐associated genes significantly colocalized with retained histones, only a handful of DMRs were situated near genes that encode miRNAs, with no significant impact to miRNA expression in APA sperm.

Both APA sperm and blastocyst DMRs exhibited significant enrichment for neurodevelopmental genes associated with autism spectrum disorder, schizophrenia, and bipolar disorder. The incidence of these neuropsychiatric conditions is known to increase progressively with increasing paternal age (de Kluiver et al., 2017), likewise DNA methylation alterations have been associated with these disorders (Rutten & Mill, 2009; Wockner et al., 2014; Wong et al., 2014). Genomic imprinting is an epigenetic phenomenon that utilizes DNA methylation to restrict gene expression to only one inherited allele. Imprinted genes play a critical role in brain development and therefore may contribute to the etiologies of neurodevelopmental conditions (Crespi, 2008). Many imprinting disorders present with autistic‐like characteristics, including Beckwith–Wiedemann syndrome (chr11p15.5), Angelman syndrome, and Prader–Willi syndrome (chr15q11‐13) (Crespi, 2008). Given that imprinting is established in the germline and persists through offspring by escaping widespread epigenetic reprogramming, it is reasonable to consider its involvement in mediating APA effects. In fact, advanced paternal age was associated with methylation differences in brain‐expressed imprinted loci in a mouse model, with concurrent behavioral changes (Smith et al., 2013). Both APA datasets comprised of various human‐imprinted genes. *DLGAP2*, which has been implicated in the development of autism (Nardone et al., 2014; Rasmussen, Rasmussen, & Silahtaroglu, 2017; Soler et al., 2018), was significantly hypomethylated in both our APA sperm and blastocyst groups. In addition, the cytoband classically responsible for Beckwith–Wiedemann syndrome (chr11p15.5) was significantly impacted in the APA sperm as well as the overlapping group of DMR‐associated genes. Hypomethylation at *KCNQ1* was statistically significant in APA sperm and blastocysts and was validated in our APA sperm samples, in accordance with previously observed hypomethylation in aged human sperm (Jenkins et al., 2014). Future studies will further examine the relationship between advanced paternal age and genomic imprinting.

Neurodevelopmental disorders in offspring may also manifest as epigenetic errors at genes associated with neurological development and function, including the opioid signaling pathway, in APA sperm. Opioid signaling was identified as a primary pathway affected in APA sperm and blastocysts, as well as in the combined directional overlapping DMRs, and shared by all three candidate gene lists for autism spectrum disorder, schizophrenia, and bipolar disorder. The frontal cortex in autistic brains was also significantly enriched for genes in the opioid signaling pathway and significantly overlapped our APA sperm and blastocyst DMR‐associated gene lists (Nardone et al., 2014). The opioid signaling pathway in the brain is a neurotransmitter system involved in mood regulation, and abnormal brain opioid activity likely plays a role in the genesis of neurodevelopmental disorders. Increased opioid receptors are reported in autism and schizophrenia cases (Pellissier, Gandia, Laboute, Becker, & Le Merrer, 2018; Volk, Radchenkova, Walker, Sengupta, & Lewis, 2012), and there is a strong similarity between symptoms of opiate addiction and autistic symptoms (Kalat, 1978). Conversely, autistic‐like symptoms have shown to be attenuated by opioid blocking in the severely mentally disabled (Sandman et al., 1983). These contrary reactions suggest that a delicate balance is required in the opioid signaling pathway, where either excess or deficiency may produce autistic‐like symptoms (Pellissier et al., 2018).

*CACNA1H* is one gene identified in the opioid signaling pathway. It encodes CaV3.2, a T‐type calcium channel abundantly expressed in the brain and implicated in neuronal function and brain development. It interacts with opioid receptors in the opioid signaling pathway to mediate analgesia (Altier & Zamponi, 2008), and missense mutations were identified in individuals with autism spectrum disorder (Splawski et al., 2006). Hypomethylation at *CACNA1H* was previously observed in aged human sperm compared with the same individual when he was young (Jenkins et al., 2014). Not only was aberrant DMR hypomethylation confirmed for this same gene in our APA sperm methylome and validation data, we also reveal DMR hypomethylation in the preimplantation blastocyst methylome, which we validated in embryos derived from APA fathers. Evidently some level of methylation retention is required for *CACNA1H* in blastocysts, which is then lost in those derived from APA fathers. Altered CaV3.2 calcium channel activity could ultimately lead to affected neuronal function and brain development in these offspring.

DNA methylation during spermatogenesis is susceptible to errors that can be propagated to the subsequent generation. It is important to note that while a large proportion of the sperm ejaculate can be analyzed for epigenetic alterations, only one methylation pattern from a single sperm utilized in fertilization has potential inheritance into the blastocyst. Current technologies do not facilitate analysis of DNA methylation in this individual sperm that requires viability. Additional limitations include the minute amount of starting material in blastocysts, the complex epigenetic reprogramming events occurring in preimplantation embryos comprising of global DNA demethylation and variable timing of remethylation based on blastocyst developmental stage and tissue lineage, and the presence of the maternal contribution from the oocyte that complicates the interpretation of epigenetic origins.

APA is a subtle and varying effect on the human population, and likewise, neurodevelopmental disorders exist on a spectrum due to substantial ranges of symptom severity. Not every child that is born to an APA father is autistic. As such, it would be unreasonable to expect a drastic alteration, similar to a gene mutation or knockout, in sperm DNA methylation in all APA fathers or derived APA blastocysts. We therefore conclude that a threshold for cumulative risk may exist in terms of aberrant epigenetic alterations in sperm that, if surpassed, culminates in a predisposition to disease and ultimately an observed phenotype in offspring. Our data substantiate an increasingly compromised DNA methylation profile as sperm ages. For the first time, we also demonstrate a generational correlation in sperm and embryo of an altered human methylation landscape associated with APA, with significant susceptibility at genes associated with neurodevelopmental disorders. Ongoing investigation into the critical window of sperm epigenetic reprogramming will further our understanding of the role of paternal age in the etiology of these inherited neurological conditions.

## 4 Experimental Procedures

### 4.1 Patient selection

Advanced paternal age (APA) was defined as male age ≥ 50 years, and young control paternal age (young) was defined as male age ≤ 35 years. Sperm and blastocysts were divided into these two groups based on paternal age at the time of oocyte retrieval. Patient demographics and cycle outcomes can be found in Table S1. Surplus sperm donated with patient consent (*n* = 36) met normozoospermic standards based on semen parameter analysis including concentration, motility, and morphology (Kruger strict), DNA fragmentation and were primarily nonsmokers. Samples were prepared by a two‐layer (90%–45%) PureSperm density‐gradient centrifugation (Nidacon) with subsequent swim‐up technique to collect the viable progressively motile portion. A somatic cell lysis step (0.1% SDS, 0.5% Triton X‐100 in H_2_O) removed round cell contamination, and clean motile sperm samples were diluted to 5 × 10^6^ sperm and stored at − 80°C in lysis buffer (Norgen Biotek).

Surplus cryopreserved blastocysts (*n* = 36) were donated from couples (*n* = 19) undergoing IVF treatment, with informed consent and Institutional Review Board approval. Only young fertile donor oocyte IVF cycles with normozoospermic male partners and a successful live birth were included. Blastocysts included in the study were scored based on developmental stage, inner cell mass, and trophectoderm appearance (Gardner & Schoolcraft, 1999), and were all morphologically graded as high, transferrable quality (grade ≥ 3BB). Ovarian stimulation, oocyte retrieval, intracytoplasmic sperm injection (ICSI), embryo culture, vitrification, and warming procedures were routinely performed as previously reported (Schoolcraft & Katz‐Jaffe, 2013).

### 4.2 Global bisulfite sequencing (WGBS or RRBS)

Individual sperm samples (*n* = 12) underwent RRBS with the use of the Methyl Mini‐Seq platform (Zymo Research). Samples underwent digestion with 60 units of TaqαI and 30 units of MspI (New England Biolabs) sequentially, and addition of adapter sequences was followed by bisulfite treatment using the EZ DNA Methylation‐Lightning Kit (Zymo Research) and purification (DNA Clean & Concentrator‐5; Zymo Research). Individual blastocysts (*n* = 12) underwent WGBS with the use of the Methyl Maxi‐Seq platform (Zymo Research). Bisulfite conversion was performed using the EZ DNA Methylation‐Direct Kit (Zymo Research) with subsequent amplification steps to add adapter and barcode sequences and PCR purification (DNA Clean & Concentrator‐5; Zymo Research). All samples underwent sequencing on the Illumina HiSeq 2,500 platform. Sequence reads were identified with the use of standard Illumina base‐calling software with a 5x minimum read count filter and theoretical resolution for detection at 20%. Bisulfite sequence data alignments were performed using the alignment software Bismark between APA and young groups. Index files were mapped to the human genome (hg19). Methylation percentage was calculated as the number of reads reporting a C, divided by the total number of reads reporting a C or T. Statistical significance of the methylation difference was determined using the Student's *t* test (sperm) or Fisher's exact test (blastocyst), where *p* < .05 was deemed to be significant. Promoter and gene body annotations were included where available. CpG island annotations were downloaded from the UCSC genome browser hg19. CpG shores were defined as those areas within 2kb on either side of an island, and shelves were defined as areas within 2kb of either side of a shore.

### 4.3 Differentially methylated region (DMR) analysis

Differentially methylated regions were identified in R (v3.5.1) using DMRcate (v1.18.0) (Peters et al., 2015) and significantly differentially methylated CpGs (*p* ≤ .05). DMRs were called using lambda = 1,000 and kernel adjustment C = 75. Due to the interdependence of the significance of p‐values in a sliding window analysis, additional filtering parameters were implemented following window identification; DMRs were required to have ≥ 3 significant CpGs and a mean |%methdiff| ≥10%. DMRs with a difference in methylation ≥ 10% in the APA samples were considered hypermethylated and ≤‐10% were considered hypomethylated. Directional DMR‐associated genes had at least one DMR in a shared methylation direction between sperm and blastocyst datasets. For unsupervised hierarchical clustering, significant DMR‐associated CpGs (*p* ≤ .05) were clustered using Pearson's rank correlation and average linkage using the “hclust” function with the pheatmap package in R.

### 4.4 Targeted bisulfite pyrosequencing for methylation validation

Individual sperm (*n* = 36) and blastocysts (*n* = 24) underwent targeted bisulfite pyrosequencing using the PyroMark Q24 Advanced system (Qiagen). Genomic DNA was isolated using the Norgen RNA/DNA/Protein Kit (Norgen) for sperm and the QIAamp DNA Micro Kit (Qiagen) for blastocysts. Bisulfite conversion was performed using the EZ DNA Methylation‐Direct Kit (Zymo Research), followed by nested PCR amplification with the use of AmpliTaq Gold (Applied Biosystems) and a universal reverse biotinylated primer in the second round. Primers for methylation analysis (CpG) were designed with the use of PyroMark Assay Design Software v.2.0.1.15 (Qiagen) and overlapped ≥ 2 significant CpGs from the originally identified DMR. Pyrosequencing reactions were prepared using the PyroMark Q24 Advanced CpG Kit (Qiagen), and the DNA methylation level was calculated as a ratio of the C to T peaks at a given CpG site using PyroMark Q24 Advanced Software v.3.0.0. (Qiagen). Student's *t* test and Mann–Whitney U test were used for methylation differences between young and APA cohorts, where *p* ≤ .05 was considered to be statistically significant. Simple linear regression was used to calculate the association between APA and methylation as age increased. Models with *p* ≤ .05 were considered significant. Pyrosequencing primers designed in‐house are outlined in Table S9.

### 4.5 Kernel density estimate

All genes from hg19 RefSeq annotations were localized by promoter start site. All genes and DMR‐associated genes were individually parsed by chromosome. For density analysis, start sites per chromosome were smoothed using a Gaussian kernel and a 0.2 bandwidth adjustment from the “density” function in R. A 95% confidence interval was generated by bootstrapping. Random sampling with replacement of the original data points followed by density estimate was performed 10,000 times to calculate a bootstrap variance. For cytoband enrichment, start sites were mapped to hg19 cytoband locations downloaded from the UCSC genome browser. Enrichment was tested using a Fisher exact test where p‐values were adjusted for multiple comparison using the Benjamini–Hochberg method and *p* ≤ .05 were considered significant.

### 4.6 Pathway analysis

DMR‐associated genes were used as input for core analysis in Ingenuity Pathway Analysis (IPA; version 1–13) (Qiagen). Gene enrichment analyses were performed in R using Fisher's exact test and a *p* ≤ .05 to define significance.

### 4.7 Public datasets

Autism spectrum disorder (ASD) genes were downloaded from SFARI Gene (https://gene.sfari.org/, accessed 10/11/18), a database of genes implicated in autism susceptibility. Genes were filtered for an enrichment score ≤ 3. Schizophrenia (SZ) genes were downloaded from a database (http://www.szdb.org/download.php, accessed 8/13/19) collated from two genome‐wide association studies (Pardinas et al., 2018; Schizophrenia Working Group of the Psychiatric Genomics, 2014) and from a database of differentially methylated genes (http://www.szdb.org/methy.php, accessed 11/20/18). Bipolar disorder (BD) genes were downloaded from a database reported in the literature (http://bdgene.psych.ac.cn/directSearch.do?type=gene&keyword=, accessed 10/15/18) and were filtered for genes positively associated with BD in ≥ 1 study.

Processed microarray β‐values for GSE53924 (Nardone et al., 2014) were downloaded from the NCBI GEO website. This dataset consists of Illumina HumanMethylation450 BeadChip methylation profiles for autistic and nonautistic individual brain samples from the frontal cortex and the cingulate cortex. A *t* test was used to calculate significant differences between groups. Probes with *p* ≤ .05 were processed through our DMR pipeline to test for regional enrichment with the only difference that DMRs were required to have ≥ 2 significant CpGs. A threshold of *p* ≤ .01 was used to compare significant pathways.

The current list of known and putative human‐imprinted genes was downloaded from the Genomic Imprinting Website (http://www.geneimprint.com, accessed 10/03/2019) and used to determine overlap of our APA sperm and blastocyst DMR‐associated genes with imprinted genes.

### 4.8 Small RNA sequencing and analysis

Individual sperm samples (*n* = 12) underwent small RNA sequencing to assess the miRNA expression profiles between young and APA. Paired‐end libraries were constructed with the Small RNA‐Seq Library Prep Kit (Lexogen) and sequenced 150 cycles on an Illumina NovaSEQ6000. Reads were trimmed for adapters, quality, and minimum length of 15 bases using bbduk.sh (BBMap v38.75). Reads ≤ 31 bases were retained by Cutadapt v1.9.1. Reads were aligned to GRCh38 (GSNAP v2019‐09–12), filtered (SAMtools v1.9) and counted (Subread featureCounts v1.6.2 with miRBase v22.1 mature miRNAs). Differential expression was calculated in edgeR v3.24.3 using Fisher's exact test. Adjusted p‐values (Benjamini–Hochberg) with a false discovery rate (FDR) ≤5% were considered significant.

## CONFLICTS OF INTEREST

The authors have declared that no conflict of interest exists.

## AUTHOR CONTRIBUTIONS

M.M.D. contributed to study design, executed critical analyses, performed methylation validation experiments, and took the lead in manuscript preparation. M.E.H. executed critical analyses, bioinformatics, and pathway analyses, and participated in manuscript preparation. J.C.P collected and processed samples for all experiments, and participated in manuscript preparation. W.B.S. provided financial support, sample contribution, and clinical input. M.G.K‐J designed and supervised the completion of the study, and participated in critical discussion and in manuscript preparation.

## Supporting information

Supplementary Material

## Data Availability

The data that support the findings of this study are available from the corresponding author upon reasonable request.
