# Supplementary material for: The inherited methylome landscape is directly altered with paternal aging and associated with offspring neurodevelopmental disorders
Source: Aging Cell. 2020 Jul 1;19(8):e13178. doi: 10.1111/acel.13178 (PMC7431824; doi:10.1111/acel.13178)
Supplement: Supplementary file 1 — Supplementary Material [file ACEL-19-e13178-s001.docx]

**Supplementary Table 1)** Patient demographics and normal range semen parameters for sperm and blastocyst samples

| **Parameters** | **Young** | **APA** |
| --- | --- | --- |
| **Ages** | | |
| Paternal Age | 30.1 ± 2.1 | 55.1 ± 3.4 ** |
| Maternal Age (Blastocysts) | Young fertile donor oocyte | Young fertile donor oocyte |
| **Semen parameters and male patient demographics** | | |
| Sperm Count | 89.9 ± 54.2 | 109.4 ± 65.4 |
| Sperm Motility | 62% ± 18% | 58% ± 14% |
| Sperm Morphology | 3.0% ± 1.5% | 3.1% ± 1.6% |
| Total Motile Count | 247.8 ± 224.1 | 141.7 ± 158.8 |
| DNA Fragmentation | 2.4% ± 2.7% | 4.1% ± 2.9% |
| Paternal BMI | 29.2 ± 6.0 | 26.7 ± 4.1 |
| Paternal Smoking Status | 71% Never | 92% Never |
|  | 24% Quit | 8% Quit |
|  | 5% Current | 0% Current |
| **IVF cycle data** | | |
| Pregnancy outcomes | 85% Live Birth | 75% Live Birth |
|  | 3% Miscarriage | 8% Miscarriage |
|  | 0% Biochemical | 4% Biochemical |
|  | 12% No Transfer | 13% No Transfer |

** p<0.0001

**Supplementary Table 2)** Methylome read mapping statistics

| **Samples** | **Total Read Number** | **Mapping Efficiency** | **Unique CpGs** | **Average CpG Coverage** | **Bisulfite Conversion Rate** | **Gene Body Coverage** | **Promoter Coverage** | **CpG Island Coverage** |
| --- | --- | --- | --- | --- | --- | --- | --- | --- |
| **Sperm (Methyl-MiniSeq)** | | | | | | | | |
| YNG 1 | 30,919,771 | 47% | 9,717,284 | 8X | 99% | 85% | 74% | 77% |
| YNG 2 | 33,860,516 | 46% | 9,864,863 | 8X | 99% | 85% | 74% | 79% |
| YNG 3 | 33,330,722 | 46% | 10,355,505 | 8X | 99% | 85% | 75% | 80% |
| YNG 4 | 32,212,578 | 42% | 10,145,132 | 7X | 99% | 85% | 74% | 78% |
| YNG 5 | 32,023,879 | 47% | 9,912,019 | 8X | 99% | 85% | 74% | 78% |
| YNG 6 | 32,236,741 | 48% | 9,837,071 | 8X | 99% | 85% | 75% | 79% |
| APA 1 | 31,247,009 | 47% | 10,129,874 | 8X | 99% | 85% | 75% | 79% |
| APA 2 | 32,567,449 | 45% | 10,220,222 | 8X | 99% | 85% | 75% | 79% |
| APA 3 | 32,471,117 | 42% | 9,873,598 | 7X | 99% | 85% | 74% | 77% |
| APA 4 | 30,202,170 | 43% | 9,663,748 | 7X | 99% | 85% | 73% | 77% |
| APA 5 | 31,611,911 | 45% | 9,816,449 | 8X | 99% | 85% | 74% | 77% |
| APA 6 | 35,743,721 | 48% | 10,036,958 | 9X | 99% | 85% | 76% | 81% |
| **Blastocysts (Methyl-MaxiSeq)** | | | | | | | | |
| YNG 1 | 422,906,276 | 62% | 22,896,016 | 30X | 99% | 85% | 69% | 56% |
| YNG 2 | 484,339,064 | 61% | 28,370,405 | 30X | 99% | 86% | 71% | 60% |
| YNG 3 | 462,453,232 | 61% | 43,593,680 | 17X | 99% | 89% | 84% | 75% |
| YNG 4 | 481,752,559 | 64% | 29,478,372 | 31X | 98% | 87% | 76% | 67% |
| YNG 5 | 487,212,124 | 62% | 36,443,768 | 23X | 99% | 88% | 81% | 71% |
| YNG 6 | 489,702,951 | 63% | 36,094,786 | 24X | 98% | 88% | 77% | 68% |
| APA 1 | 502,958,355 | 61% | 42,018,862 | 21X | 99% | 89% | 83% | 78% |
| APA 2 | 507,076,029 | 63% | 22,090,645 | 40X | 99% | 86% | 71% | 58% |
| APA 3 | 510,044,505 | 62% | 38,726,517 | 23X | 98% | 88% | 76% | 66% |
| APA 4 | 466,725,178 | 61% | 38,469,550 | 20X | 98% | 89% | 81% | 70% |
| APA 5 | 448,954,497 | 60% | 38,299,371 | 19X | 99% | 88% | 80% | 68% |
| APA 6 | 495,812,948 | 63% | 37,621,620 | 25X | 99% | 87% | 74% | 64% |

**Supplementary Figure 1)** Global DNA methylation in Young and APA sperm

*


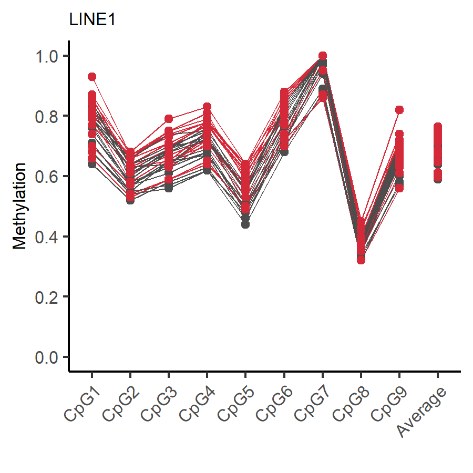


*


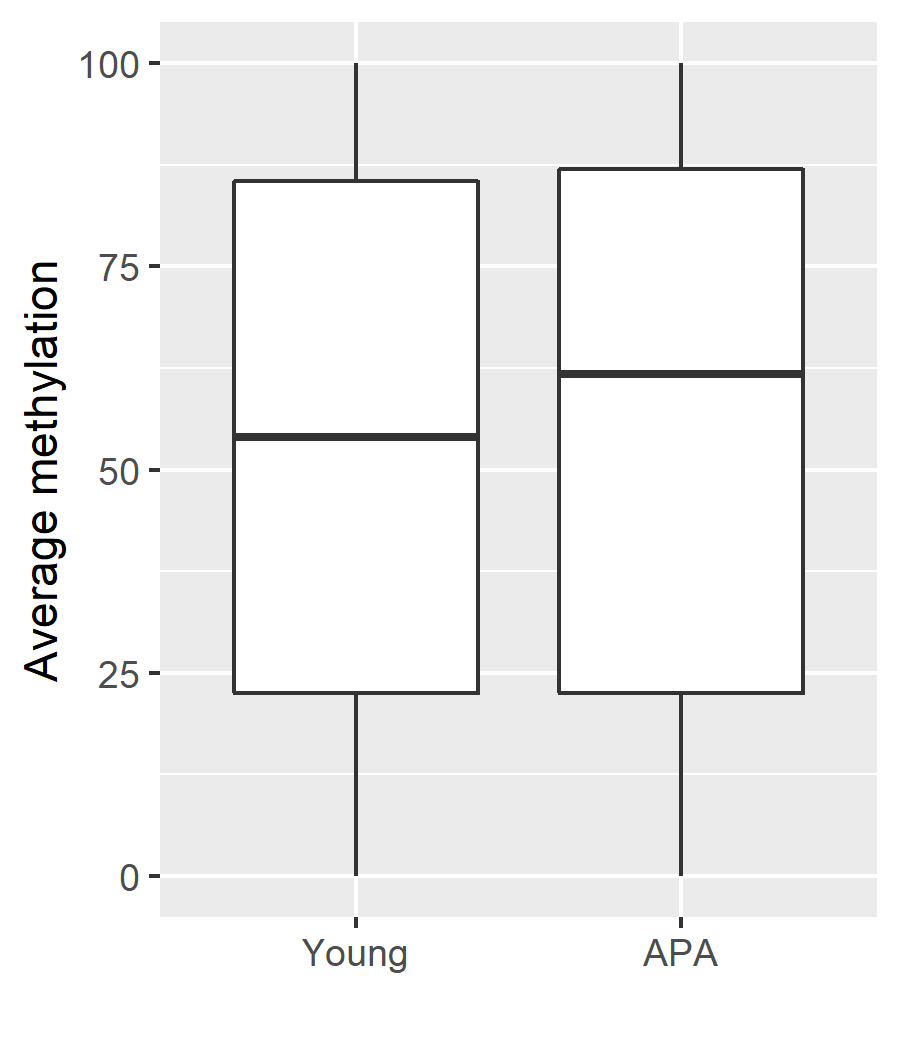


A

B

Global DNA methylation in Young and APA sperm. A) The distribution of the average Young and APA means for significant sperm CpGs visualized using boxplots. The bottom and top of each box represent the 1st and 3rd quantiles of the distributions, and the line between represents the median. The distributions are significantly different according to a Mann-Whitney test (*p=1.33x10^-21^).

B) LINE1 bisulfite pyrosequencing. CpG sites on the x-axis are followed by the average percent methylation across all CpGs for Young (gray) and APA (red) individuals. Each line represents results for individual sperm samples (n=18 Young and n=18 APA sperm samples). Significant hypermethylation is observed in APA (70%) relative to Young (67%), *p<0.05.

**Supplementary Table 3)** DMR-associated genes previously identified in aging human sperm (Jenkins et al. 2014)

| **Gene** | **Methylation Status** | **Association** |
| --- | --- | --- |
| AGRN | Hypomethylated |  |
| ARC | Hypomethylated |  |
| ATHL1 | Hypomethylated |  |
| BEGAIN | Hypomethylated |  |
| C7orf50 | Hypomethylated |  |
| CACNA1H | Hypomethylated | Autism Spectrum Disorder, Opioid Signaling |
| CCDC114 | Hypomethylated |  |
| DAPK3 | Hypomethylated |  |
| DLGAP2 | Hypomethylated | Imprinted |
| DRD4 | Hypomethylated | Bipolar Disorder |
| ELANE | Hypomethylated |  |
| FOXK1 | Hypomethylated |  |
| GET4 | Hypomethylated | Schizophrenia |
| GRIN1 | Hypomethylated | Autism Spectrum Disorder, Bipolar Disorder, Opioid Signaling |
| HOXA10 | Hypomethylated |  |
| KCNA7 | Hypomethylated |  |
| KCNF1 | Hypomethylated |  |
| KCNQ1 | Hypomethylated | Imprinted |
| KDM2B | Hypomethylated |  |
| LDLRAD4 | Hypomethylated |  |
| LONP1 | Hypomethylated |  |
| MPPED1 | Hypomethylated |  |
| NADK | Hypomethylated |  |
| NCOR2 | Hypomethylated | Schizophrenia, Frontal Cortex of Autistic Brains |
| NSMF | Hypomethylated |  |
| PALM | Hypomethylated |  |
| PAX2 | Hypomethylated |  |
| PITPNM1 | Hypomethylated |  |
| PTPRN2 | Hypomethylated | Frontal Cortex of Autistic Brains |
| PURA | Hypomethylated |  |
| SECTM1 | Hypomethylated |  |
| SLC22A18AS | Hypomethylated | Schizophrenia, Imprinted |
| SOHLH1 | Hypomethylated |  |
| THBS3 | Hypomethylated |  |
| UNKL | Hypomethylated | Schizophrenia |
| USP36 | Hypomethylated |  |
| WFDC1 | Hypomethylated |  |
| ZFPM1 | Hypomethylated | Schizophrenia |
| BCL11A | Hypermethylated | Autism Spectrum Disorder, Bipolar Disorder |
| CCDC144NL | Hypermethylated |  |
| FAM86C1 | Hypermethylated |  |

*Jenkins, T. G. et al. Age-associated sperm DNA methylation alterations: possible implications in offspring disease susceptibility. PLoS Genet, 10(7), e1004458. doi:10.1371/journal.pgen.1004458 (2014)

**Supplementary Figure 2)** Sperm and blastocyst DNA methylation validation results for *CACNA1H* and *SHANK2*


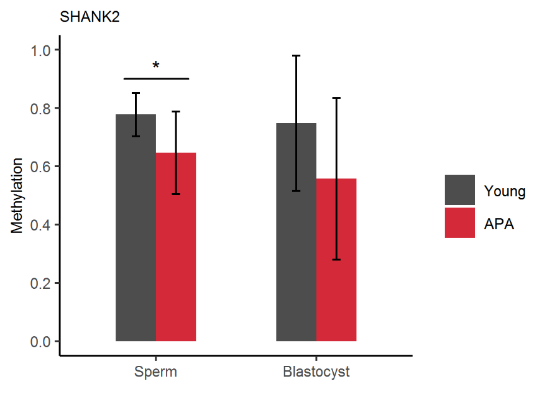

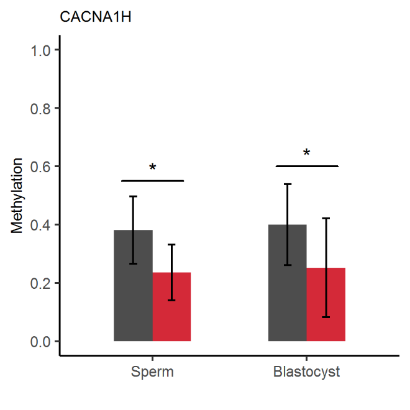


B

A

Sperm and blastocyst DNA methylation validation results for CACNA1H and SHANK2. Bar plots showing the average methylation changes within a significant DMR for two selected validation genes, (A) CACNA1H and (B) SHANK2, in sperm (n=12) and blastocyst (n=12) for Young (grey) and APA (red) fathers. Error bars represent one standard deviation. Significant hypomethylation is demonstrated for both sperm and blastocyst in CACNA1H and for sperm in SHANK2. *p≤0.05

**Supplementary Table 4)** Statistically significant cytobands

| **Cytoband** | **APA Sample** | **P-value** | **FDR** | **Odds Ratio** |
| --- | --- | --- | --- | --- |
| Chr10q26.3 | Sperm | 1.79E-04 | 2.91E-02 |  |
|  | Blastocyst | 3.93E-05 | 2.66E-03 |  |
|  | Overlapping | 2.25E-02 | N.S. | 5.29 |
| Chr11p15.5 | Sperm | 6.95E-08 | 2.45E-05 |  |
|  | Blastocyst | 4.92E-02 | N.S. |  |
|  | Overlapping | 2.98E-04 | 3.03E-02 | 5.95 |
| Chr16p13.3 | Sperm | 9.02E-08 | 2.45E-05 |  |
|  | Blastocyst | 2.43E-07 | 3.81E-05 |  |
|  | Overlapping | 6.50E-05 | 1.05E-02 | 4.20 |
| Chr17q25.3 | Sperm | 7.52E-05 | 1.53E-02 |  |
|  | Blastocyst | 2.81E-07 | 3.81E-05 |  |
|  | Overlapping | 6.59E-03 | N.S. | 3.80 |
| Chr19p13.3 | Sperm | 4.32E-19 | 3.51E-16 |  |
|  | Blastocyst | 3.76E-22 | 3.06E-19 |  |
|  | Overlapping | 5.62E-16 | 4.57E-13 | 9.89 |

**Supplementary Table 5)** Co-localization of DMR-associated genes with histones and histone modifications

| **Histone gene set*** | **Overlapping genes** | **Fold enrichment (odds ratio)** | **OR 95% confidence interval** | **P-value** |
| --- | --- | --- | --- | --- |
| **APA Sperm DMRs** | | | | |
| Mononucleosomes | 619 | 1.89 | 1.69-2.11 | 9.36E-29 |
| H3K27me3 | 275 | 1.38 | 1.20-1.59 | 7.21E-06 |
| H3K4me3 | 541 | 0.94 | 0.84-1.05 | N.S. |
| **APA Blastocyst DMRs** | | | | |
| Mononucleosomes | 1074 | 2.19 | 2.00-2.39 | 1.50E-66 |
| H3K27me3 | 364 | 1.05 | 0.93-1.18 | N.S. |
| H3K4me3 | 957 | 1.08 | 0.98-1.18 | N.S. |
| **APA Sperm-Blastocyst Directional Overlapping DMRs** | | | | |
| Mononucleosomes | 130 | 3.32 | 2.51-4.41 | 2.52E-18 |
| H3K27me3 | 31 | 0.90 | 0.59-1.32 | N.S. |
| H3K4me3 | 79 | 0.82 | 0.62-1.10 | N.S. |

*Hammoud, S. S. et al. Distinctive chromatin in human sperm packages genes for embryo development. Nature 460, 473-478, doi:10.1038/nature08162 (2009).

**Supplementary Table 6)** Top canonical pathways for neurodevelopmental disorders

| **Autism Spectrum Disorder** | **P-value** | **Schizophrenia** | **P-value** | **Bipolar Disorder** | **P-value** |
| --- | --- | --- | --- | --- | --- |
| GABA Receptor Signaling | 2.19E-08 | Synaptic Long Term Depression | 2.29E-06 | Serotonin Receptor Signaling | 1.26E-23 |
| CREB Signaling in Neurons | 4.90E-07 | Synaptic Long Term Potentiation | 6.03E-06 | Circadian Rhythm Signaling | 2.51E-23 |
| Glutamate Receptor Signaling | 5.62E-06 | Dopamine-DARPP32 Feedback in cAMP Signaling | 1.86E-05 | CREB Signaling in Neurons | 2.51E-19 |
| Calcium Signaling | 4.68E-05 | p70S6K Signaling | 7.76E-05 | G-Protein Coupled Receptor Signaling | 6.31E-18 |
| Opioid Signaling Pathway | 5.25E-05 | CREB Signaling in Neurons | 9.12E-05 | GABA Receptor Signaling | 3.98E-17 |
| Amyotrophic Lateral Sclerosis Signaling | 6.46E-05 | Calcium Signaling | 1.95E-04 | Dopamine Receptor Signaling | 5.01E-17 |
| GPCR-Mediated Nutrient Sensing in Enteroendocrine Cells | 6.76E-05 | Opioid Signaling Pathway | 1.05E-03 | cAMP-mediated signaling | 3.16E-15 |
| nNOS Signaling in Skeletal Muscle Cells | 1.32E-04 | D-myo-inositol-5-phosphate Metabolism | 1.23E-03 | Opioid Signaling Pathway | 3.98E-15 |
| Corticotropin Releasing Hormone Signaling | 3.02E-04 | 14-3-3-mediated Signaling | 1.38E-03 | Calcium Signaling | 6.31E-15 |
| Synaptic Long Term Depression | 3.72E-04 | Role of NFAT in Cardiac Hypertrophy | 1.48E-03 | Neuroinflammation Signaling Pathway | 3.16E-14 |

**Supplementary Table 7)** DMR-associated imprinted genes

| **APA Sample** | **DMR** | **Chr** | **Imprinted Gene** | **Imprinting Status** | **Expressed Allele** |
| --- | --- | --- | --- | --- | --- |
| Sperm | Hypomethylated | chr1 | DVL1 | Predicted | Maternal |
| Blastocyst | Hypomethylated/Hypermethylated | chr1 | OBSCN | Predicted | Paternal |
| Blastocyst | Hypermethylated | chr1 | PEX10 | Predicted | Maternal |
| Sperm | Hypomethylated/Hypermethylated | chr1 | PRDM16 | Predicted | Paternal |
| Blastocyst | Hypomethylated | chr1 | PRDM16 | Predicted | Paternal |
| Sperm | Hypermethylated | chr1 | PTPN14 | Predicted | Maternal |
| Sperm | Hypomethylated | chr1 | TP73 | Imprinted | Maternal |
| Blastocyst | Hypomethylated | chr2 | GPR1 | Imprinted | Paternal |
| Blastocyst | Hypomethylated | chr2 | MYEOV2 | Predicted | Paternal |
| Blastocyst | Hypomethylated | chr3 | ALDH1L1 | Predicted | Maternal |
| Blastocyst | Hypermethylated | chr6 | IGF2R | Conflicting Data | Biallelic |
| Blastocyst | Hypomethylated/Hypermethylated | chr6 | PRIM2 | Conflicting Data | Biallelic |
| Blastocyst | Hypomethylated | chr6 | ADTRP | Imprinted | Maternal |
| Sperm | Hypomethylated | chr7 | DDC | Imprinted | Isoform Dependent |
| Blastocyst | Hypomethylated | chr7 | HOXA3 | Predicted | Maternal |
| Sperm | Hypermethylated | chr7 | MAGI2 | Imprinted | Maternal |
| Blastocyst | Hypomethylated | chr7 | SLC4A2 | Predicted | Maternal |
| Sperm | Hypomethylated | chr8 | DLGAP2 | Imprinted | Paternal |
| Blastocyst | Hypomethylated | chr8 | DLGAP2 | Imprinted | Paternal |
| Blastocyst | Hypomethylated | chr8 | ZFAT | Imprinted | Paternal |
| Sperm | Hypomethylated | chr9 | EGFL7 | Predicted | Paternal |
| Sperm | Hypomethylated | chr10 | VENTX | Predicted | Maternal |
| Sperm | Hypomethylated | chr11 | ANO1 | Imprinted | Maternal |
| Sperm | Hypomethylated | chr11 | B4GALNT4 | Predicted | Maternal |
| Blastocyst | Hypomethylated | chr11 | B4GALNT4 | Predicted | Maternal |
| Sperm | Hypomethylated | chr11 | H19 | Imprinted | Maternal |
| Sperm | Hypomethylated | chr11 | IGF2;INS-IGF2 | Imprinted | Paternal |
| Sperm | Hypomethylated | chr11 | KCNQ1 | Imprinted | Maternal |
| Blastocyst | Hypomethylated/Hypermethylated | chr11 | KCNQ1 | Imprinted | Maternal |
| Blastocyst | Hypomethylated | chr11 | NAP1L4 | Unknown | Unknown |
| Blastocyst | Hypomethylated | chr11 | NTM | Imprinted | Maternal |
| Blastocyst | Hypomethylated | chr11 | OSBPL5 | Imprinted | Maternal |
| Blastocyst | Hypomethylated | chr11 | RAB1B | Predicted | Maternal |
| Sperm | Hypomethylated | chr11 | SLC22A18AS | Provisional Data | Maternal |
| Sperm | Hypermethylated | chr11 | WT1 | Imprinted | Paternal |
| Sperm | Hypomethylated | chr12 | FBRSL1 | Predicted | Maternal |
| Blastocyst | Hypomethylated/Hypermethylated | chr12 | FBRSL1 | Predicted | Maternal |
| Sperm | Hypermethylated | chr12 | LRP1 | Imprinted | Unknown |
| Sperm | Hypomethylated | chr14 | DLK1 | Imprinted | Paternal |
| Sperm | Hypomethylated | chr14 | RTL1 | Imprinted | Paternal |
| Sperm | Hypermethylated | chr15 | GABRG3 | Conflicting Data | Paternal |
| Blastocyst | Hypomethylated | chr15 | SNRPN | Imprinted | Paternal |
| Blastocyst | Hypomethylated | chr16 | NAA60 | Imprinted | Maternal |
| Sperm | Hypomethylated | chr16 | SOX8 | Predicted | Paternal |
| Blastocyst | Hypermethylated | chr19 | DNMT1 | Imprinted | Paternal |
| Sperm | Hypermethylated | chr19 | LILRB4 | Predicted | Maternal |
| Blastocyst | Hypermethylated | chr19 | NLRP2 | Imprinted | Maternal |
| Blastocyst | Hypermethylated | chr19 | PEG3;ZIM2;MIMT1 | Imprinted | Paternal |
| Sperm | Hypomethylated | chr19 | PPAP2C | Predicted | Maternal |
| Blastocyst | Hypermethylated | chr19 | ZNF229 | Predicted | Maternal |
| Blastocyst | Hypomethylated | chr20 | GNAS | Imprinted | Isoform Dependent |
| Sperm | Hypomethylated | chr20 | HM13 | Unknown | Unknown |
| Blastocyst | Hypomethylated | chr20 | HM13 | Unknown | Unknown |

**Supplementary Figure 3)** Heatmap of miRNAs expressed in sperm


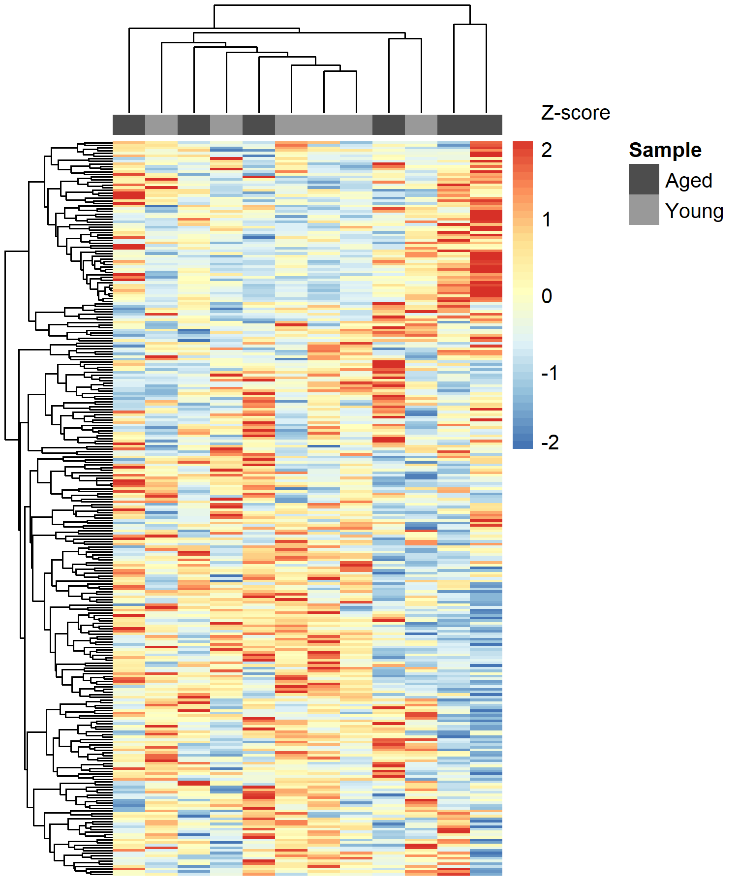


Small RNA-sequencing analysis revealed no differentially expressed miRNAs in aged compared to young sperm (p≤0.05, q≤0.05). Hierarchical clustering, where a positive z-score corresponds to increased expression and a negative z-score corresponds to decreased expression relative to the mean, demonstrates similar expression patterns across all samples.

**Supplementary Table 8)** DMRs in APA sperm that co-localize with genes encoding miRNAs

| **DMR Location** | **Methylation Status** | **Promoter** | **Exon** | **Intron** |
| --- | --- | --- | --- | --- |
| chr10:122557687-122558147 | hypomethylated |  |  | WDR11-AS1;MIR5694 |
| chr10:135186002-135187041 | hypomethylated | ECHS1;MIR3944 |  | ECHS1 |
| chr11:2154157-2154854 | hypomethylated | MIR483 | IGF2;INS-IGF2 |  |
| chr13:36133868-36133992 | hypermethylated |  |  | NBEA;MIR548F5 |
| chr14:48229894-48229966 | hypermethylated | MIR548Y |  |  |
| chr14:101347799-101347933 | hypomethylated | MIR433;MIR431 | RTL1 |  |
| chr15:69220522-69220562 | hypomethylated |  |  | MIR548H4 |
| chr16:2139894-2140277 | hypomethylated | MIR1225 | PKD1;MIR1225 | PKD1 |
| chr16:22734301-22734740 | hypomethylated |  |  | MIR548AA2;MIR548D2 |
| chr20:55923653-55924378 | hypomethylated |  |  | MIR5095 |
| chr21:28674884-28674922 | hypermethylated |  |  | MIR5009 |
| chr21:29201234-29201246 | hypermethylated |  |  | MIR5009 |
| chr22:49175623-49175861 | hypomethylated | MIR4535 |  |  |
| chr3:99717507-99717518 | hypermethylated | MIR548G |  | CMSS1;FILIP1L |
| chr8:145619128-145620171 | hypomethylated | MIR939 | CPSF1 |  |
| chr10:122557687-122558147 | hypomethylated |  |  | WDR11-AS1;MIR5694 |

**Supplementary Table 9)** Primers for targeted bisulfite pyrosequencing

| **Gene** | **Forward Primer** | **Reverse Primer** | **Sequencing Primer** | **# of CpGs (# Overlapping DMR)** |
| --- | --- | --- | --- | --- |
| *CACNA1H Sperm* | TAGTTGGGGTGGTTAGGT | CGCCAGGGTTTTCCCAGTCACGACACCCCTCCTACTACCAACCTATCT | GTTAGGTAGTGGTTTT | 6 (3) |
| *CACNA1H Blastocyst* | AGTGTTTAGGGATTTAGTTATAGATGA | CGCCAGGGTTTTCCCAGTCACGACCATACCCTAAAACCTCCCCCTTCC | AGGGGTTGTGAGTTAAA | 7 (2) |
| *CNTNAP2* | GTTTTGTATGTATAGGAAGTGATAGTTT | CGCCAGGGTTTTCCCAGTCACGACAATACAAAAAAAAAAAAACCCCAAATC | ATGTATAGGAAGTGATAGTTTT | 6 (3) |
| *COMT* | TGATATTTTATAAGAGGTGGTTGAGTAG | CGCCAGGGTTTTCCCAGTCACGACCCCCAACAAAACCACTAAAAAACTCCTTAA | ATAGGGAAAGGGAGAT | 7 (3) |
| *DRD4* | GTGAATTTAGGAGGTTGGGGTAGA | CGCCAGGGTTTTCCCAGTCACGACCAAAAAAACAAACAACCCCTCTAA | GGGGTAGAGATTAGTAGT | 10 (3) |
| *GRIN1* | AGAATTTGTAGGTAGGGTAGGT | CGCCAGGGTTTTCCCAGTCACGACCCCTAAAAACACTCACCACTAC | AGGTAGGGTAGGTTATT | 5 (2) |
| *KCNQ1* | GGTATTTGATAGAGGATTTGGTTTAGA | CGCCAGGGTTTTCCCAGTCACGACAATTTCACCACCTTAACCAAACTAATCTCT | TTGGTTTAGAATATAGAAATATGTA | 5 (4) |
| *LINE1* | GATTTTTAGAGTTAGGTGTGGGATATAGT | CGCCAGGGTTTTCCCAGTCACGACAAAATCAAAAAATTCCCTTTCTAAATCA | AGTTAGGTGTGGGATATAGT | Modified from Urdinguio (2015)* |
| *MBP* | AGTATTAAAGAGGTTGAAGAGAGTTATT | CGCCAGGGTTTTCCCAGTCACGACAACCAAAACCTCACACAATATCTAT | ATTTTTATTTTTAAAAAATAGTAGT | 5 (3) |
| *PRKCZ* | GGGGGGTGGAAAGGAAAATAG | CGCCAGGGTTTTCCCAGTCACGACCCCCTCCTCACATAATCCTCATAATATC | AAAATAGGTAGGGGATAG | 9 (4) |
| *SHANK2 Sperm* | AGATAGGGTGGAGATGTTAGAT | CGCCAGGGTTTTCCCAGTCACGACAAACCAACCAATCTTTCAAACTAT | GGTGGAGATAGTGTTG | 9 (4) |
| *SHANK2 Blastocyst* | AGGAGTTAGAGATTAGTTTGGTTAATATGA | CGCCAGGGTTTTCCCAGTCACGACAAAATACACAAAAACCCCTACTT | GTTTGGTTAATATGATATTTGTAAT | 4 (3) |
| *SHANK3* | AGGGAAAAAGTTGGGAGAAAG | CGCCAGGGTTTTCCCAGTCACGACATCATACTACCTCATCTACACTT | GTTTTTGGGAGGGAAT | 6 (2) |
| *TCF3* | AGGGGAGAGTGTTTGTTGAAT | CGCCAGGGTTTTCCCAGTCACGACCCCCTAAAAACTCAATTTCCCCATCT | AGTTGTTTGGATGGGA | 5 (4) |
| *TRPM2* | AGGGAATATTGGTTTGTGATTTATTGA | CGCCAGGGTTTTCCCAGTCACGACACAAAACCCAACAACTAATCC | GGTGATTTATTTTTTTGATAGTG | 5 (2) |
| *ZNF804A* | TGAGAAAGGGTGAGAAATGATAGAAGA | CGCCAGGGTTTTCCCAGTCACGACCAAAAATTCCAAACCAACCTAAC | GTTGGAGTGTAGTGG | 7 (3) |
|  |  | **Biotin Universal Reverse Primer** |  |  |
|  |  | 5'Biotin-CGCCAGGGTTTTCCCAGTCACGAC |  |  |

*Urdinguio, R. G., Bayon, G. F., Dmitrijeva, M., Torano, E. G., Bravo, C., Fraga, M. F., . . . Fernandez, A. F. (2015). Aberrant DNA methylation patterns of spermatozoa in men with unexplained infertility. Hum Reprod, 30(5), 1014-1028. doi:10.1093/humrep/dev053
